# Vesicle-Templated Self-Assembly of Programmable Freestanding Multi-μm DNA Shells

**DOI:** 10.1021/acs.nanolett.6c00402

**Published:** 2026-05-14

**Authors:** Hao Yuan Yang, Christoph Karfusehr, Friedrich C. Simmel

**Affiliations:** † Department of Bioscience, TUM School of Natural Sciences, Technical University of Munich, Am Coulombwall 4a, 85748 Garching, Germany; ‡ Max Planck School Matter to Life, Jahnstraße 29, D-69120 Heidelberg, Germany

**Keywords:** DNA nanotechnology, self-assembly, synthetic biology, compartmentalization

## Abstract

In the quest to create increasingly complex synthetic cell-mimicking systems, diverse DNA nanostructures have been developed to coat, permeabilize, sculpt, or otherwise functionalize lipid vesicles or used as scaffolds to direct vesicle growth. Here, we introduce a simple, broadly applicable method to realize freestanding, membrane-mimicking DNA shells: DNA shells are first assembled on giant unilamellar vesicles and then liberated by surfactant-mediated liposome removal, retaining the geometry of their membrane templates. We demonstrate this approach using two distinct DNA tecton classes: a complex barrel-shaped DNA origami and a simple 11-oligonucleotide nanostar-inspired motif. The site-specific addressability of DNA origami structures enables the rational design of binding interfaces, as demonstrated by the controlled formation of multilayer shells. The success of both strategies underscores the feasibility of using different DNA architectures to create tunable, DNA-only shell-like compartments spanning the size range of eukaryotic cells, thereby offering a fundamentally new type of compartmentalization for bottom-up synthetic biology.

## Graphical abstract

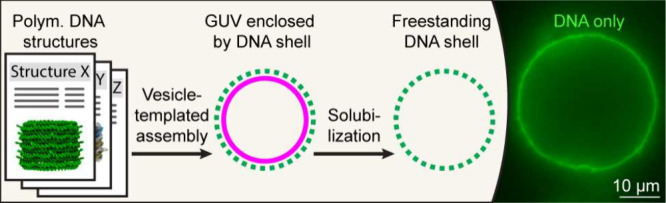

Compartmentalization is one of the hallmarks of living systems, where it fulfills a wide range of essential roles. It provides dedicated environments for specific metabolic processes, separates cooperative from parasitic interactions, and prevents the loss of reactants by diffusion, among others. For the same reasons, compartmentalization is also regarded as a prerequisite for the synthetic realization of complex life-like assemblies. Alongside membraneless compartments based on liquid–liquid phase separation, cells typically employ lipid bilayer membranes for compartmentalization. Inspired by this principle, bottom-up synthetic biology has frequently adopted giant unilamellar vesicles (GUVs) as chassis for realizing functional modules that act as synthetic organelles or even entire synthetic cells.^1–4^ However, one of the major challenges for this approach is that loading hydrophilic cargo into the lumen of preassembled GUVs or exchanging materials with the environment requires specialized transport systems, which often limits the implementation of more complex chemical functions.^5^

Over the past decade, DNA nanotechnology has provided a versatile toolset for constructing functional modules within synthetic cellular systems. These modules can be used to controllably manipulate membrane architectures and to recapitulate many of the biological functions typically carried out by membrane-associated proteins. The versatility of DNA-based assemblies results from their precisely tunable mechanical properties, rich structural diversity, and site-specific addressability that allows stoichiometrically defined and spatially precise placement of molecules.^6–8^ In particular, advances in chemical conjugation strategies^9,10^ have made it possible to attach hydrophobic moieties to defined sites on oligomeric DNA or DNA origami structures, thereby enabling controlled interactions with lipid membranes.^11^

DNA origami structures have been interfaced with lipid membranes and vesicles for the formation of artificial nanopores,^12–14^ to facilitate lipid transfer,^15^ induce membrane deformation or sculpting,^16–18^ membrane budding,^19^ and membrane fusion.^20^ Lipid bilayers have been employed to promote the growth of DNA origami lattices by restricting their assembly to two dimensions.^21^ DNA nanofilaments have further been used as artificial cytoskeletons, or cell cortices for synthetic cell models based on vesicles,^22–24^ droplets,^25^ or membraneless phase-separated compartments.^26,27^ Conversely, DNA origami structures of various sizes and shapes have been used as scaffolds for the assembly of lipid nanosheets^28^ and lipid vesicles of defined sizes^29^ or morphologies,^30,31^ as well as the production of lipid-bilayer nanodiscs.^32^

Previously, liquid–liquid phase-separating DNA nanostars have been used to form a patterned DNA hydrogel layer inside GUVs, which can be released upon surfactant treatment to yield freestanding DNA gel capsules.^33^ Such an approach requires the encapsulation of relatively large (μM) DNA concentrations, and the use of cationic lipids to promote electrostatic binding complicates applications involving negatively charged cargoes. Furthermore, the resulting capsule walls form disordered, gel-like structures, which are not amenable to precise and programmable modifications, such as functionalization of the inner and outer layers. Once released, the capsules often adopt distorted rather than well-defined spherical shapes, which further affects the positions of any attached functional components. Here we demonstrate an alternative approach in which more complex DNA origami units selectively bind and assemble on the outer surface of lipid bilayer templates, bypassing the need for monomer encapsulation and enabling controlled shell growth prior to release. This strategy yields freestanding multi-μm shells with a well-defined local structure and a preserved, template-guided shape.

We first developed a membrane-forming system based on a rigid, precisely addressable membrane-forming DNA origami monomer that can bind to GUV surfaces mediated via cholesterol linkers. This barrel-shaped monomer termed “Dipid” has weak isotropic inter-origami interactions and can mimic lipid membrane assembly, enabling the formation of closed containers reaching the size of *E. coli* cells, multi-μm planar membrane-like sheets, while supporting the integration of diverse functional modules.^34^ Subsequent removal of the lipid template via surfactant treatment yielded freestanding multi-μm Dipid membrane structures. We also show that the cholesterol-mediated outer-leaflet-templated assembly approach enables the formation of freestanding GUV-sized containers based on a minimal monomer system composed of only 11 DNA strands per subunit, which robustly retain the shape of their templates.

We defined two criteria for suitable shell-forming monomers: (i) the ability to polymerize into contiguous shells and (ii) the capacity to bind lipid membranes via hybridization with DNA strands conjugated to hydrophobic moieties. These requirements are fulfilled by our Dipid monomers, which are barrel-shaped, hollow structures with a diameter of 30 nm^34,35^ (Figure 1a-c). Dipids assemble into multi-μm monolayers through 30 binding strands extending from the barrel surface^34^ (Figure 1a,d). As shown in the figure, each binding strand consists of two distinct subdomains. The sticky domains carry self-complementary sequences that promote binding to other Dipids, largely independent of angular orientation. As the sequences of strands in different layers around the cylindrical body are distinct, they enforce monomer assembly in a single axial orientation. The flex domains, on the other hand, are composed exclusively of thymidines, providing a means to modulate the mechanical properties of the shell (Figure 1b). For example, the estimated dimer pitch bending modulus decreases approximately 8-fold from *B*_pitch_ = 49 *k*_*B*_ *T* for 2T flex domains to *B*_pitch_ = 6 *k*_*B*_ *T* for 16T flex domains (Figure S1). We denote Dipid designs as “PxT”, where *x* refers to the number of T’s in the flex domain, and five different designs were tested (*x* = 2, 4, 8, 16, 32). For incorporation into lipid membranes, Dipid monomers were equipped with three single-stranded DNA handles protruding from the bottom side of the barrel. These hybridize to linker oligonucleotides carrying a 5′ cholesterol moiety, which are inserted into the GUV membranes (Figure 1a).

**Figure 1:**
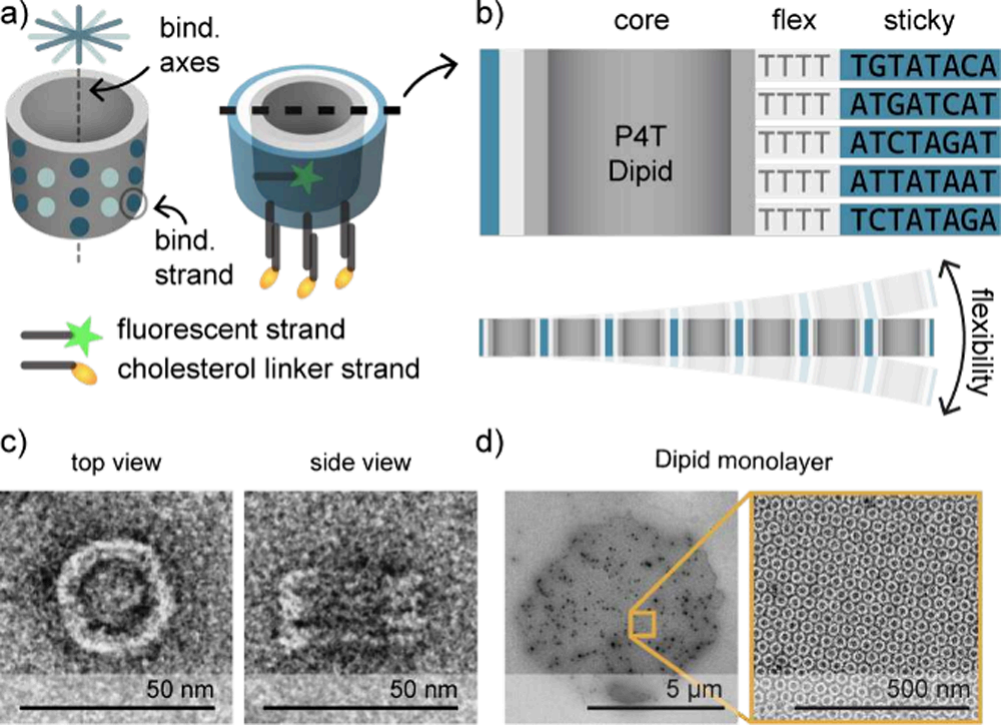
Design of Dipids: programmable and well-structured shell-forming DNA origami monomers. a) Left: Dipids are barrel-shaped, hollow monomers constructed from a core barrel (gray) featuring 30 ssDNA binding strands (blue spots), distributed on two 6-fold axes on the barrel’s outer surface, approximating isotropic interactions. Right: Each binding strand consists of a flex domain encoding shell flexibility (light gray) and a sticky domain encoding controlled polymerization (blue). Each Dipid is also functionalized with a fluorophore (green star) conjugated to an internal DNA strand as well as with ssDNA handles (dark gray rods) extending from the bottom of the barrel that bind to linker strands conjugated to cholesterol (yellow ellipses). b) Top: Cross-section of a P4T Dipid showing the sequences of the flex and sticky domains. Bottom: A sheet of assembled Dipids schematically showing the flexibility of Dipid membranes. c) Negative stain transmission electron microscopy (TEM) micrographs showing top and side views of the Dipid monomer. d) Negative stain TEM micrographs showing a multi-μm assembled Dipid monolayer (P16T) next to a zoom-in.

Non-templated Dipid monomers readily self-assemble into large 3D aggregates during the initial folding step (Figure 1d). To reassemble Dipid shells on GUV templates, these aggregates must first be disassembled into monomers by heating above a disassembly temperature specific to each Dipid variant. Hence, we performed dynamic light scattering (DLS) measurements for each Dipid variant during both heating and cooling in stepwise increments of Δ*T* = 1 K, with the half-decay lag time (τ_1/2_) extracted at each step (Figure 2a and S2). During heating, a sudden decrease in τ_1/2_ indicated rapid disassembly of Dipid aggregates into smaller fragments and monomers leading to a large spread in τ_1/2_ values, whereas during subsequent cooling, a gradual increase in τ_1/2_ reflected slower monomer reassembly, consistent with previous work on DNA origami assembly and disassembly.^36^

**Figure 2:**
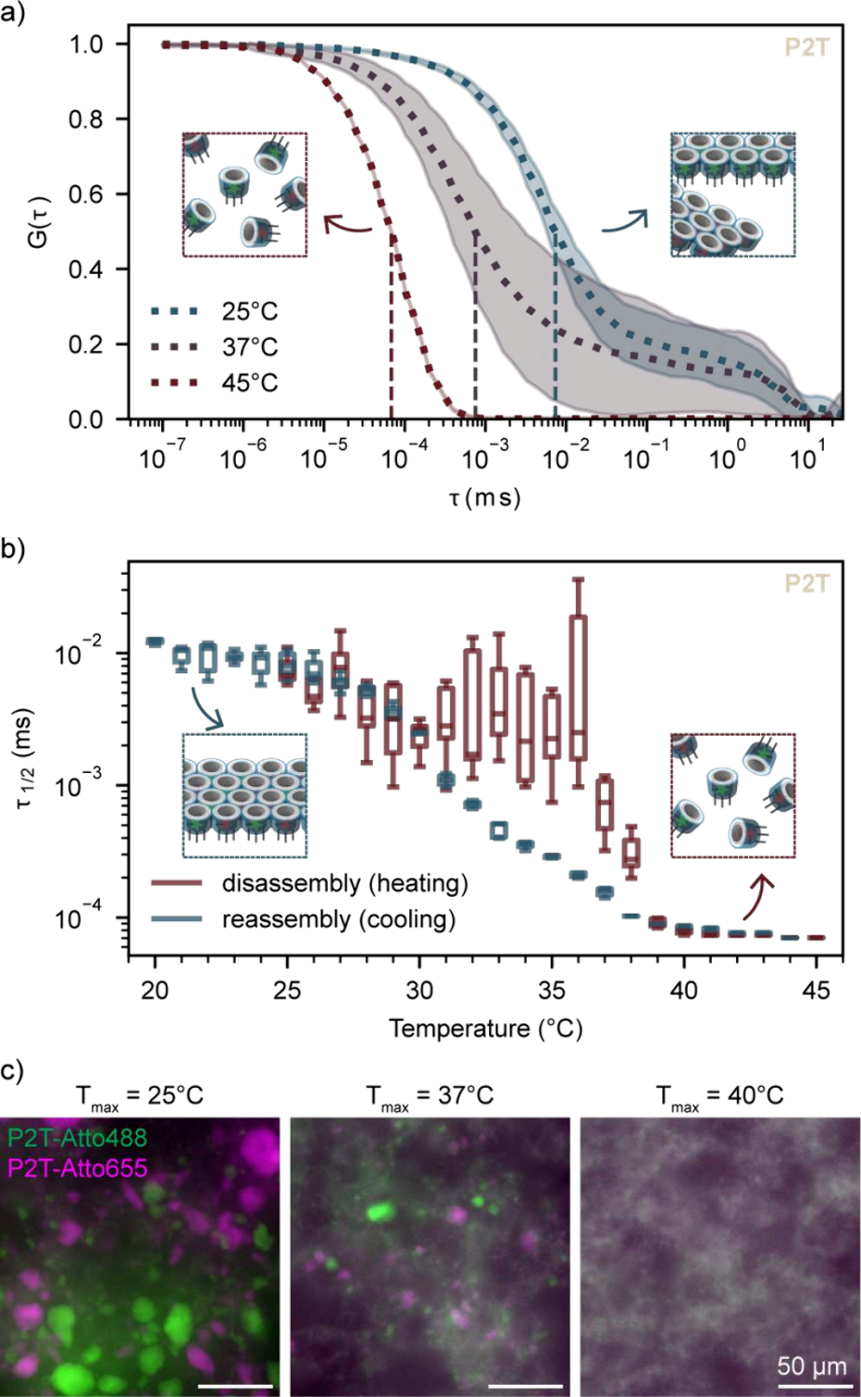
Determination of disassembly temperature. a) Normalized autocorrelation curves of P2T via DLS upon heating up to 25 °C (inset showing Dipid aggregates containing different fluorescently labeled monomers prior to disassembly), 37 °C, and 45 °C (inset showing disassembled Dipid monomers). Curves represent the statistical mean of *n* = 10 measurements for each acquisition temperature; shaded areas represent the standard deviation of the same data, and vertical dotted lines indicate the half-decay lag times, τ_1/2_. b) Extracted τ_1/2_ of P2T at each temperature step upon disassembly and reassembly via gradual heating (inset showing disassembled Dipid monomers) and cooling (inset showing reassembled Dipid sheets containing a mix of both Dipid monomers). Boxes show medians and interquartile ranges (IQR); whiskers extend to 1.5 × IQR. c) Fluorescence microscopy images showing morphology of P2T upon gradual cooling after sample incubation at the respective temperatures *T*_max_. Scale bars: 50 μm.

The experiments indicated nearly complete disassembly of all variants at 40 °C (Figure 2b and S3). Accordingly, 40 °C was generally used to disassemble Dipid aggregates prior to their reassembly on GUVs. Notably, variants with longer flex domains (P16T and P32T) required lower disassembly temperatures, which may be attributed to the greater entropic contribution of the flexible linkers.

We further confirmed the disassembly and reassembly protocol by fluorescence microscopy. To this end, we mixed two differently labeled samples of the same Dipid variant (labeled with Atto488 or Atto655) and heated them to a defined maximum temperature, *T*_max_. The samples were then gradually cooled to allow liberated monomers to reassemble. Mixed structures containing both labels were expected only if disassembly had occurred during the heating step. As expected, after heating to *T*_max_ = 40 °C all Dipid variants appeared fully mixed, indicating complete disassembly. Heating to intermediate *T*_max_ resulted in both mixed structures and smaller chunks of aggregates showing partial disassembly, in agreement with our DLS data (Figure 2c and S4).

Figure 3 illustrates the step-by-step production of freestanding multi-μm DNA shells. To this end, we first separately generated GUVs via electroformation, as well as folded and purified Dipid structures of any variant (P2T is shown as an example). Both GUVs and Dipids were then combined such that Dipid monomers are in excess in a solution composition that prevents unspecific DNA-lipid interactions^17^ and reduces GUV sedimentation (Step 1, Figure 3, see also Methods).

**Figure 3:**
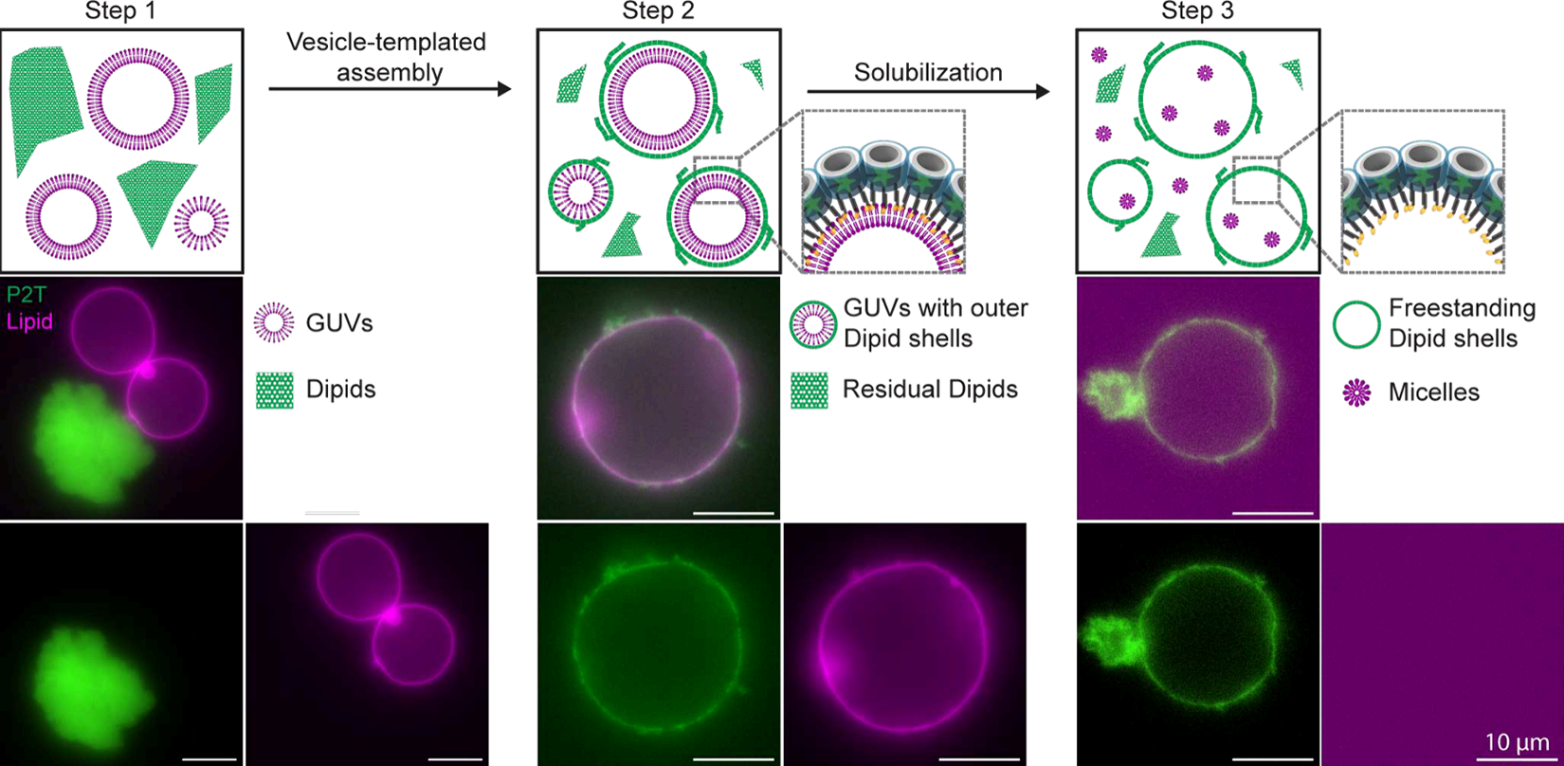
Formation of freestanding DNA shells using the Dipid framework. Schematic illustration of the three-step strategy for the formation of Dipid-based freestanding DNA shells, with accompanying fluorescence images. Step 1: A solution containing GUVs (magenta) and excess Dipids (green) is prepared in a composition where unspecific interaction and GUV sedimentation are reduced (see Methods). Step 2: Heating the solution at 40 °C for 1 h induces Dipid disassembly, and gradual cooling by – 0.1 °C every 3 min facilitates vesicle-templated assembly of Dipids, forming GUVs enclosed by an outer Dipid shell. Step 3: Upon surfactant treatment using 0.1% Triton X–100, GUVs are solubilized into micelles, resulting in freestanding multi-μm DNA shells. Scale bars: 10 μm.

Subsequently, the Dipid structures were disassembled by heating to 40 °C. During gradual cooling, the monomers reassembled on the surface of GUVs and GUV aggregates, facilitated by hybridization of their handles with membrane-anchored cholesterol linkers, thereby forming an outer Dipid shell (Step 2, Figure 3 and S5). The fuzzy appearance of the shell likely reflects Dipid monolayer outgrowths, previously observed in self-assembled Dipid membrane clusters.^34^ These outgrowths are seeded by origami dimers or other misfolded structures and could, in principle, be suppressed by improved monomer purification.

Following solubilization of the GUVs into micelles by addition of the surfactant Triton X–100, we obtained freestanding multi-μm DNA shells where the lipid signal is now delocalized (Step 3, Figure 3 and S6). The resulting shells appeared contiguous (Figure S7). However, during solubilization, some DNA shells collapsed. As the Dipids were linked to the GUVs via cholesterol-binding handles, the forces generated through rapid liposome dissolution are transmitted to the DNA shells. Notably, we observed a higher tendency of collapse for shells formed by P32T monomers, which may be due to their higher linker flexibility (Figure S6).

We additionally demonstrated that each component of the Dipid monomer design is required for correct assembly. The absence of cholesterol-binding handles or leaving the reaction at room temperature without first inducing Dipid disassembly prevented shell formation (Figure S8a,b). On the other hand, the absence of sticky domains in the monomer (“P8Td”) did not result in the formation of freestanding Dipid shells after solubilization (Figure S8c).

Cryo-ET structural studies have shown that Dipids indeed self-assemble into monolayers as designed.^34^ We therefore surmised that templated growth of Dipids would also occur in monolayers. To test this hypothesis, we compared the fluorescence signal of labeled Dipids on GUV templates with that of monolayers formed by non-polymerizing P8Td Dipids (Figure 4a,b). Compared to the P8Td monolayer control, the median Dipid shell intensity is ∼1.2 times higher and exhibits a broader distribution (*I*_P8T_ = 3547 ± 1689 au, *I*_P8Td_ = 3026 ± 950 au). This likely reflects additional intensity contributions from membrane outgrowths, suggesting that Dipids are mostly organized in monolayers.

**Figure 4:**
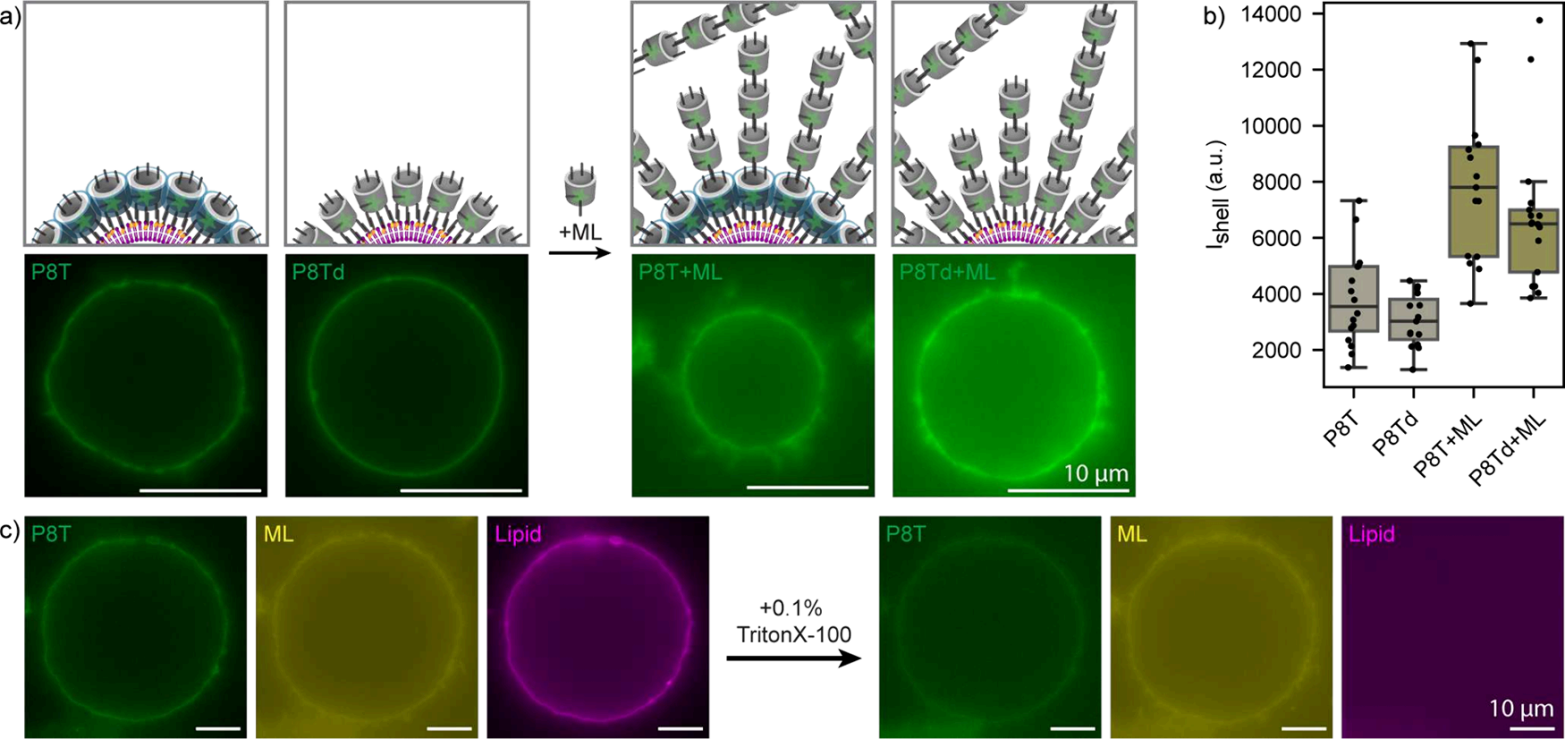
Formation and release of multilayer shells. a) Schematics showing comparison of P8T Dipid shells (green) with non-polymerizing P8Td Dipid shells (green) assembled on GUVs before and after addition of a multilayer Dipid variant (“ML”, green) capable of radial polymerization. Corresponding fluorescence microscopy images were recorded under identical conditions and are shown with the same contrast settings, with a representative image shown. b) Median background-corrected fluorescence intensities of Dipid layers of analyzed templated structures. Boxes show medians and interquartile ranges (IQR); whiskers span 1.5 × IQR. Sample sizes: *n*_P8T_ = 16, *n*_P8Td_ = 15, *n*_P8T+ML_ = 15, and *n*_P8Td+ML_ = 17. Comparison of the shell-forming P8T and non-polymerizing P8Td structures suggests that the P8T Dipids indeed assembled into a monolayer. c) Fluorescence microscopy images of a P8T DNA shell (green) assembled on a GUV (magenta) with a multilayer Dipid shell on the outside (yellow) before and after release using Triton X–100. Scale bars: 10 μm.

Biological membranes exhibit high structural diversity, ranging from single phospholipid bilayers to complex multilamellar systems.^37^ To realize analogous architectures with our Dipids, we exploited the site-specific addressability of DNA origami structures. In this approach, an initial monolayer serves as a template for the stepwise construction of more complex multilayer shells. Notably, exposure of the outer surface of the first layer to the surrounding solution provides direct access for the implementation of layer-specific modifications. As a proof of principle, we developed a multilayer (“ML”) Dipid that binds to the top of shell Dipids and polymerizes top-to-bottom with copies of itself (Figure 4a and Figure S9). The addition of ML Dipids to preformed Dipid shells increased shell thickness, evident from increased shell intensities (Figure 4a,b, *I*_P8T+ML_ = 2.2 × *I*_*P*8T_, *I*_P8Td+ML_ = 1.8 × *I*_P8Td_). Releasing such multilayer structures produced our largest freestanding Dipid shells (diameter ≈ 40 μm) (Figure 4c and Figure S10, S11), which notably retained the size and shape of their GUV templates (Figure 4c). If finer layer control is desired, the Dipid shell system enables stepwise addition of individual Dipid layers carrying small molecules such as fluorescent dyes (cf. Figure S9, S10, for a two-layer system) or other functional units.

We hypothesized that membrane-like structures could also be formed by other types of nucleic acid assemblies, not necessarily restricted to our approximately 140-component DNA origami Dipid design. To test whether our assembly method is applicable to less structured systems, we designed a minimal monomer inspired by a DNA nanostar with six pinned-down arms (Figures 5a and S12). This monomer consists of 11 DNA strands and assembles into a small barrel with six binding domains and an additional ssDNA loop that hybridizes to a cholesterol-modified DNA strand. We simulated the structure of the minimal monomer using oxDNA,^38^ which suggested that the designed structure can be formed experimentally.

**Figure 5:**
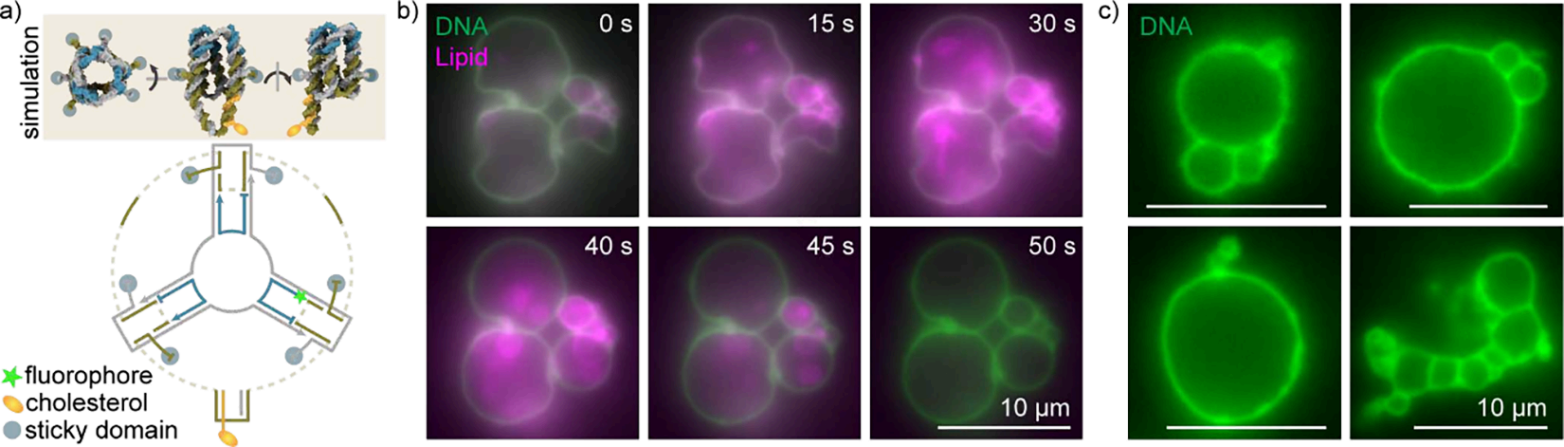
Formation of freestanding DNA shells from a minimal monomer. a) Mean structure from an oxDNA simulation (top) and scheme of the DNA strand routing of the minimal monomer (bottom). Strands are colored to highlight routing symmetries, with the placement of fluorophore (green star), cholesterol molecules (yellow ellipses), and sticky domains (blue spots) also indicated. Dotted, desaturated lines represent strand domain connections of zero length. Sticky domains have the sequence “TT GCGC” (Figure S9). b) Fluorescence microscopy time series of DNA shell (green) release from GUV templates (magenta). Triton X–100 was added to one side of a narrow channel containing GUVs enclosed by DNA shells and allowed to diffuse. *t* = 0 s marks the start of acquisition. c) Examples of freestanding DNA shells after Triton X–100 treatment. Scale bars: 10 μm.

We reasoned that the formation of a contiguous DNA shell does not strongly depend on the details of the monomer structure provided that the monomers can assemble into extended sheets with sufficient mechanical stability. We expected that our minimal monomer would yield such sheets through their symmetric and approximately isotropic intersubunit interactions, which are realized by their six self-complementary binding domains. To test this, we implemented a one-pot protocol that combined monomer folding with vesicle-templated shell assembly in a single annealing step. As expected, this yielded DNA shells assembled on both the GUVs and GUV aggregates (Figure 5b).

Upon treatment with surfactant, the GUV templates dissociated from the DNA shells, passing through intermediates that included tubular structures and smaller liposomes, and ultimately leaving freestanding DNA shells (Figure 5b,c) with no detectable lipid signal at the DNA boundary above the background signal (Figure S13a,b). Notably, we sometimes observed deformed templated structures that relaxed into spherical shells after liposome destruction, thus retaining the sizes and shapes of the GUV templates (Figure 5b).

In summary, we have developed a straightforward and versatile strategy to create freestanding, eukaryotic-cell-sized DNA shells by assembling DNA nanostructures on the outer surfaces of lipid vesicles, followed by their release via surfactant treatment. Templated self-assembly on the outer GUV leaflet, mediated by hydrophobic linkers, enforces a preferred monomer orientation and enables polymerization under buffer conditions that suppress unspecific DNA-lipid interactions. Together with programmable Dipid monomer units, this produces membrane-like DNA shells with hexagonal lattice organization. The defined orientation and precise geometry of Dipids and Dipid membranes support the rational design of binding interfaces and allow the integration of functional modules as demonstrated in previous work.^34^ In addition, accessibility from the outside enables the sequential addition of functionalized shell layers.

Unlike nanostar-based gel capsules, our DNA shells retain the shapes and sizes of their lipid templates upon release. We observed distinct mechanical responses for the two monomer types used (dipid and the minimal monomer). While both types of DNA shells withstand moderate deformation, shells formed from the minimal monomer accommodate stronger template deformations and relax back to a spherical geometry after the lipid removal. We therefore anticipate that custom monomer design will enable programmable shell properties—such as membrane stiffness, porosity, and geometry retention—tailored to specific applications.

The system may be further advanced by mitigating outgrowth formation, for instance, by more extensive purification of Dipid monomers or by applying temperature-cycling and washing steps to remove unbound species. Although such outgrowths could, in principle, be exploited as high surface area features, they may interfere with downstream shell processing or with the integration of functional modules. In our experiments, designed multilayer growth yields larger freestanding DNA shells, which we rationalize to be the result of increased mechanical stability, reducing shell collapse with each additional layer. We anticipate that multilayer assembly may be further enhanced by designing different types of layer-specific dipids and supplying them to immobilized GUV templates in a microfluidic device to enable layer-by-layer assembly. Moreover, photocleavable or pH-responsive cholesterol linkers offer alternative release strategies that may further reduce the collapse during lipid removal. Together with other strategies such as silicification^39^ and UV cross-linking,^40^ the long-term stability of the shells could be enhanced.

As our strategy requires only an accessible lipid bilayer for templating, we anticipate that it may also be applicable to natural cells or organelles with complex geometries. By adding functional Dipid modules and applying the Dipid organization principles in binary mixtures developed in our previous work,^34^ we foresee that cell-sized functional DNA compartments could be realized in the near future. In the longer term, composite DNA shells incorporating biological and synthetic components may provide a route toward biohybrid compartments that support increasingly complex, programmable behaviors and could contribute to the development of biomolecular robotic systems.

## Supplementary Material



## Notes

Methods and supplementary figures, including estimates of selected mechanical properties as well as additional DLS data and fluorescence microscopy images (PDF)
