# Supplementary material for: Vesicle-Templated Self-Assembly of Programmable Freestanding Multi-μm DNA Shells
Source: Nano Lett. 2026 May 14;26(20):6560–6. doi: 10.1021/acs.nanolett.6c00402 (PMC13220298; doi:10.1021/acs.nanolett.6c00402)
Supplement: Supplementary file 1 [file nl6c00402_si_001.pdf]

Vesicle-templated self-assembly of programmable freestanding multi-μm DNA shells

Hao Yuan Yang[+,1,2], Christoph Karfusehr[+,1,2], and Friedrich C. Simmel[*1,2]

[1]Department of Bioscience, TUM School of Natural Sciences, Technical University of Munich, Am Coulombwall 4a, 85748 Garching, Germany
[2]Max Planck School Matter to Life, Jahnstraße 29, D-69120 Heidelberg, Germany
[+]These authors contributed equally.

*Email: simmel@tum.de

Supporting Information

Methods

oxDNA simulations

The nanostructure of the minimal monomer was visualized with oxView [1], which was also used to define the relaxation forces used during the subsequent oxDNA simulation. Following an initial Monte Carlo pre-relaxation, we performed molecular dynamics simulations using the oxDNA2 model [2] to relax and simulate the monomer structure. We generated PDB files for visualization in ChimeraX [3] via the oxDNA analysis tools command-line script [2]. The shown monomer structure corresponds to a mean configuration. All relevant oxDNA files are available on our GitHub repository.

DNA origami design and folding

We incorporated the modifications mentioned in the main text into the Dipid structures [4], which were in turn adapted from the DNA origami structures developed by Wickham et al. [5] using scadnano [6]. Dipids were folded with a p2873 (30 nm Dipid) scaffold (provided by Prof. Hendrik Dietz's group, 100 nM in ddH_2O) and varying sets of staple strand oligonucleotides (Integrated DNA Technologies, 200 µM in 20 mM TRIS, 0.1 mM EDTA, pH 8.0). Annotated scadnano files and all DNA sequences used in this study are available on our GitHub repository. We prepared 60 µL folding solutions containing a final scaffold concentration of 50 nM and a staple strand concentration of 200 nM in FOB18 buffer (5 mM TRIS, 1 mM EDTA, 18 mM $MgCl_2$, and 5 mM NaCl). Folding solutions were annealed in a thermocycler (Mastercycler nexus GX2, Eppendorf) using a protocol of 15 min at 65 °C followed by a decrease of 0.1 °C every 6 min from 56 °C to 53 °C.

DNA origami purification via ultrafiltration

Folded Dipid samples were purified via ultrafiltration using Amicon Ultra filters (0.5 mL, 100 K, Millipore) in 32 °C FOB5 washing buffer (5 mM TRIS, 1 mM EDTA, 5 mM $MgCl_2$, 5 mM NaCl). The filters were pre-washed using 500 µL FOB5, then loaded with 430 µL of FOB5 and 60 µL of folding solution, followed by 450 µL of FOB5, and finally 450 µL of FOB5_NaCl300 (5 mM TRIS, 1 mM EDTA, 5 mM $MgCl_2$, 300 mM NaCl), with centrifugation at 20 krcf for 5 min at 32 °C and discarding of flowthrough done at each step. The purified and buffer-exchanged samples were then extracted from the filters by repeated aspiration to dissolve any pellets.

DNA origami purification via Polyethylene Glycol (PEG) precipitation

Alternatively, we purified folded Dipid samples via PEG precipitation. Up to four folding reactions of the same Dipid type and FOB18 buffer were combined up to a volume of 750 µL. This was mixed with 750 µL of precipitation buffer (15 % *w/v* PEG 8000, 500 mM NaCl, 1x FOB0), and centrifuged at 20 krcf for 30 min at 25 °C. After removing the supernatant, and the pellet was resuspended in 750 µL of FOB18 and incubated at 30 °C, 500 rpm for 30 min. Afterwards, another 750 µL of precipitation buffer was added, and the centrifugation step was repeated. The supernatant was discarded, and the sample was resuspended in at least 50 µL of FOB5_NaCl300 by incubating at 30 °C, 500 rpm for a minimum of 30 min.

Negative stain TEM

We applied 5 µL of DNA origami solution to glow-discharged formvar carbon Cu400 TEM grids (Science Services) with a coating time of 20 s, a coating current of 35 mA, and negative polarity. The incubation time ranged from 30 to 300 s, depending on the DNA origami concentration. To prepare the staining solution, we added 1 µL of 5 M NaOH to 200 µL of 2 % uranyl formate, then vortexed and centrifuged the mixture at 21 krcf for 5 min. After sample incubation, we washed the grids with 5 µL of stain and subsequently incubated them with 15 µL of stain for 30 s. nsTEM imaging was conducted using a FEI Tecnai T12 microscope (120 kV) equipped with a Tietz TEMCAM-F416 camera and operated via SerialEM.

Dynamic light scattering (DLS)

We performed DLS measurements using a DynaPro NanoStar (Wyatt Technology), using the internal temperature control to disassemble and reassemble Dipid solutions (7.5 nM in 80 µL of FOB5_NaCl300). Dipid solutions were added to disposable MicroCuvettes (Wyatt Technology), with the chamber sealed to prevent evaporation. The same protocol was used for all Dipid variants: a slow temperature ramp of 1 °C per 10 min from 25 °C to 45 °C to 20 °C, with acquisition starting after 5 min at each step to allow the target temperature to be reached. At each temperature step, we acquired 10 acquisitions with a 30 s acquisition time and with auto-attenuation enabled. We extracted raw DLS autocorrelation curves from the instrument files, applied moving average smoothing, and performed min–max normalization of the smoothed data. For each measurement, we determined the autocorrelation time $\tau_{1/2}$, defined as the time at which $G(\tau) \leq 0.5\ G(\tau = 0)$.

GUV formation by electroformation

For all Dipid experiments, we produced GUVs by electroformation. We prepared a stock solution of 0.988% DOPC (1,2-dioleoyl-sn-glycero-3-phosphocholine, Avanti Research), 0.01% Atto655-labeled DOPE (1,2-dioleoyl-sn-glycero-3-phosphoethanolamine, ATTO-TEC), 0.002% Chol-TEG linker (oligonucleotide carrying a 5' cholesterol moiety connected via a tetraethylene glycol spacer, Biomers) in chloroform at a total concentration of 2 mM. We spread 5 µL of the solution evenly on platinum electrodes before drying in a desiccator. The electrodes were then immersed in 650 µL of 660 mM sucrose, before applying 20 Vpp at 10 Hz for 60-90 min followed by 1 Hz for another 30 min at room temperature using a custom electroformation chamber and a function generator (FG-1302, Voltcraft). We stored GUV solutions at 4 °C till further use.

GUV formation by natural swelling

For all minimal monomer experiments, we prepared GUVs by natural swelling. Specifically, we added 150 µL of 2 mM POPC (1-palmitoyl-2-oleoyl-glycero-3-phosphocholine, Avanti Research) in chloroform containing 0.5 % Atto488-labeled DOPE (ATTO-TEC) to a small round-bottom flask. We dried the lipid film using a rotary evaporator at 100 mbar for 3 h. To obtain a final lipid concentration of 100 µM, we hydrated the film with 3 mL 1 M sucrose and incubated it at 4° overnight.

Dipid shell formation

We combined 50 µL reaction solutions in a final concentration of 1.5 nM Dipids, 10% Optiprep (Serumwerk), 40 % (v/v) GUVs containing TEG-chol linkers produced via electroformation in 660 mM sucrose (final concentration of 264 mM sucrose), in 1× FOB5_NaCl300. This composition matches the osmolarity of the inner solution of the GUVs (660 mM sucrose), as determined by an osmometer (Osmomat 3000, GONOTEC GmbH), while Optiprep helps to reduce sedimentation of GUVs. The presence of 300 mM NaCl serves to prevent unspecific DNA-lipid interactions [7]. We then performed vesicle-templated assembly by incubating reaction solutions in a thermocycler using a protocol of 1 h at 40 °C followed by a decrease of 1 °C every 3 min from to 20 °C, forming GUVs with outer Dipid shells. For fluorescence imaging, 20 µL of the annealed reaction solution was added to 50 µL of imaging buffer (5 mM TRIS, 1 mM EDTA, 5 mM $MgCl_2$, 300 mM NaCl, 264 mM sucrose) into microscopy slide wells (ibiTreat µ-slide 18 well, Ibidi).

Release of Dipid shells

To release freestanding Dipid shells, we gently added 0.7 µL of Triton X–100 (1:9 diluted with FOB5_NaCl300, final 0.1%, Sigma-Aldrich) to the above-mentioned 70 µL of imaging solutions containing GUVs with outer Dipid shells.

Bilayer and multilayer Dipid shell formation and release

To form bilayer or multilayer Dipid shells, we mixed 5× excess of 1L Dipids or 25× excess of ML Dipids (pre-heated to 50 °C) respectively with solutions containing P8T Dipids assembled on GUVs in a final volume of 100 µL with the same buffer composition. The mixtures were left at room temperature for at least two

hours before the addition of 100 µL imaging buffer in microscopy slide wells (ibiTreat µ-slide 18 well, Ibidi) for fluorescence imaging. To release freestanding bilayer or multilayer Dipid shells, we similarly added 2 µL of Triton X–100 (1:9 diluted with FOB5_NaCl300, final 0.1%, Sigma-Aldrich) to the above-mentioned 200 µL of imaging solutions.

Minimal monomer shell formation

We combined 2 µL of 100 µM cholesterol strand solution (Biomers, resuspended in FOB0_NaCl500; 5 mM TRIS, 1 mM EDTA, 500 mM NaCl) with 18 µL GUV solution produced using natural swelling, and incubated the mixture for 20 min at room temperature. The one-pot folding and shell assembly mixture contained the DNA oligonucleotides (Integrated DNA Technologies, 200 µM in 20 mM TRIS, 0.1 mM EDTA, pH 8.0) "sticky1" to "sticky6", "dummy", "staple1", and "staple2" at 0.888 µM, "staple3_fluo" at 1 µM, 1× FOB_NaCl500, and 16.67 % (v/v) of the GUV solution containing cholesterol strands. We annealed the mixture in a thermocycler from 85 °C to 25 °C at $-1\,^{\circ}\mathrm{C\,h^{-1}}$.

Release of minimal monomer shells

The solution containing GUVs enclosed by minimal monomer shells was transferred to channels of a microscopy slide (µ-Slide VI 0.1, Ibidi). We added 5 µL Triton X–100 (1:100 diluted with FOB0_NaCl500) to one side of the channel and allowed it to diffuse into the channel. We recorded time series in regions where the lipid signal and GUV appearance were unchanged and waited for the surfactant to reach these positions and solubilize the GUVs. We also recorded images of freestanding DNA shells in regions with high Triton X–100 concentration and no discernible lipid vesicle signal.

Fluorescence microscopy

Each DNA monomer was designed to include one Atto488, Atto565, or Atto655-labeled DNA oligonucleotide (Biomers), while GUVs also contain either Atto488 or Atto655-labeled DOPE (ATTO-TEC). We imaged samples using a Nikon Ti-2E inverted fluorescence microscope (NIS elements software, SOLA SM II LED light source, Andor NEO 5.5 camera) with a 40× air objective (CFI Plan Fluo 40× CH, Nikon) or a 100× oil objective (Lambda D 100× Oil CFI Plan Apochrom, Nikon). Images were globally contrast-adjusted using Fiji [8].

Validation of Dipid disassembly temperatures via fluorescence microscopy

For imaging of Dipid structures after disassembly and reassembly to verify disassembly temperatures, we separately folded and purified two differently-labeled (Atto488 or Atto655) samples of the same Dipid variant, then combined them in approximately equal ratios in a final concentration of 7.5 nM in FOB5_NaCl300. The solutions were then incubated at the specified temperatures for 1 h, followed by cooling at −0.1 °C every 3 min to 20 °C in a thermocycler. Solutions were transferred into microscopy slide wells (ibiTreat µ-slide 18 well, Ibidi) prior to fluorescence microscopy imaging.

Supplementary Figures

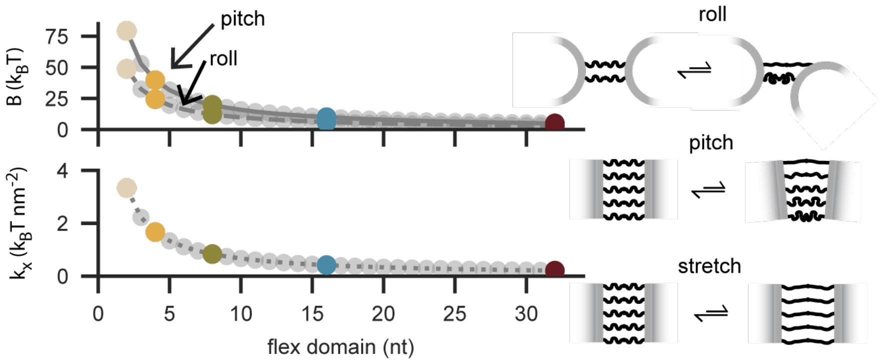

Figure S1: Estimates of selected mechanical properties of Dipid dimers. Schematics of the two bending modes and the stretching mode used to estimate the bending modulus B for each mode and the stretching modulus k_x, respectively. PxT Dipid designs used in this study are marked by colored circles. All estimates follow the framework of Videbæk *et al.* [9], adapted to Dipid geometry.

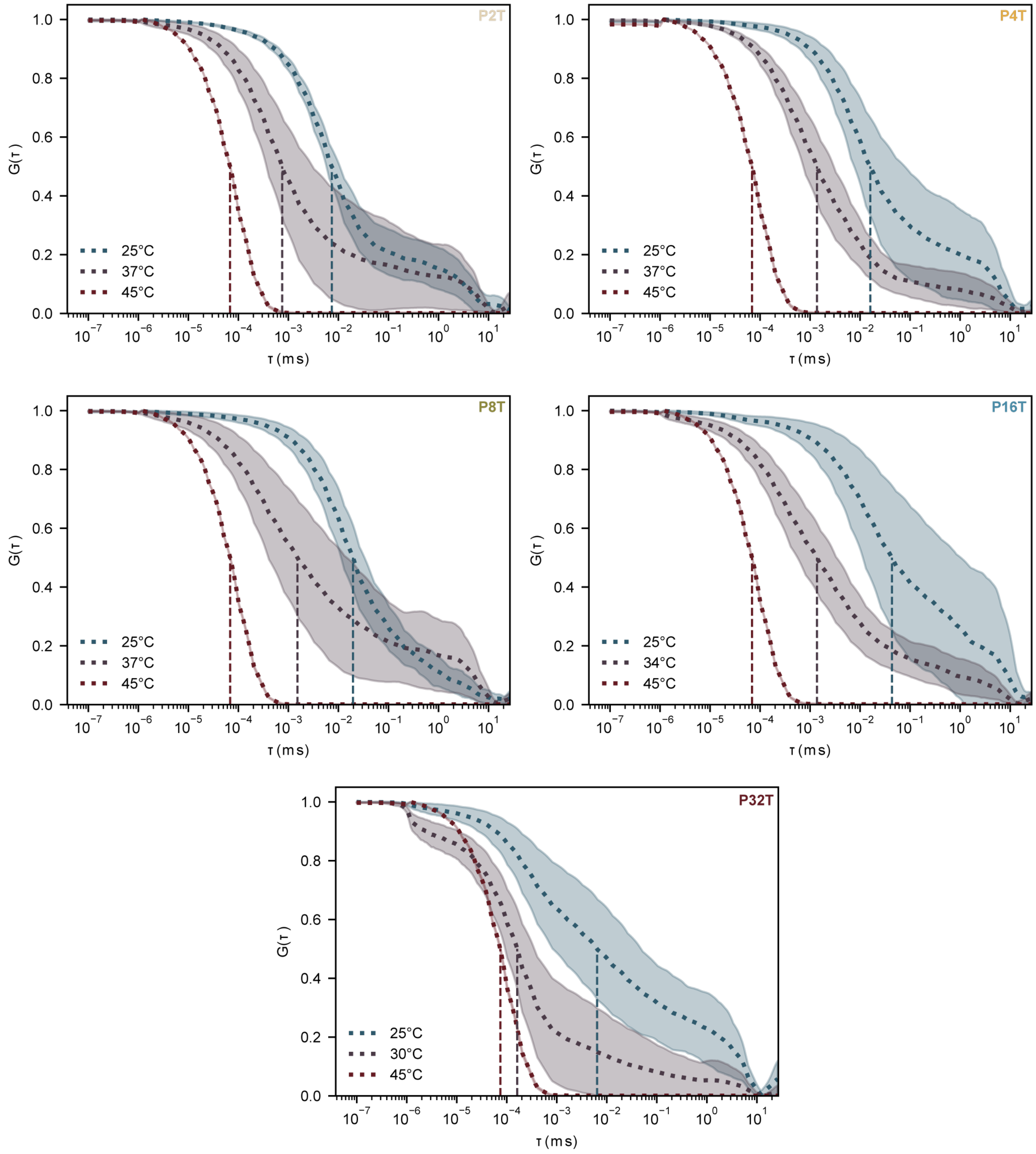

Figure S2: Normalized autocorrelation curves obtained via DLS during the disassembly of each Dipid variant at 25 °C, an intermediate temperature, and 45 °C. Curves represent the statistical mean of $n = 10$ measurements for each acquisition temperature, shaded areas represent the standard deviation of the same data, vertical dotted lines indicate the half-decay lag times, $\tau_{1/2}$.

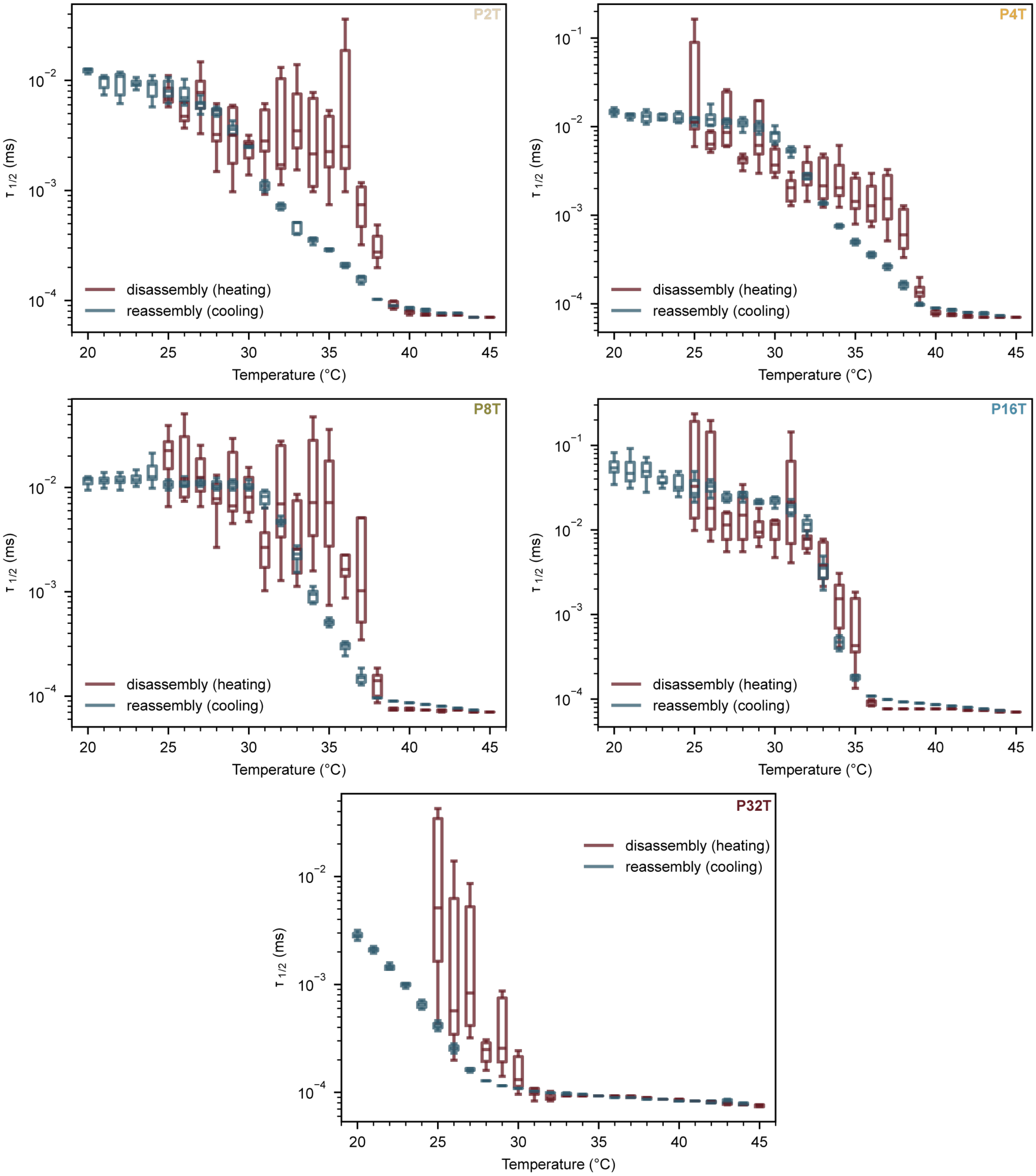

Figure S3: Extracted half-decay lag times, $\tau_{1/2}$, of each Dipid variant upon disassembly and reassembly at each temperature step obtained via DLS. Boxes show medians and interquartile ranges (IQR), whiskers extend to 1.5×IQR.

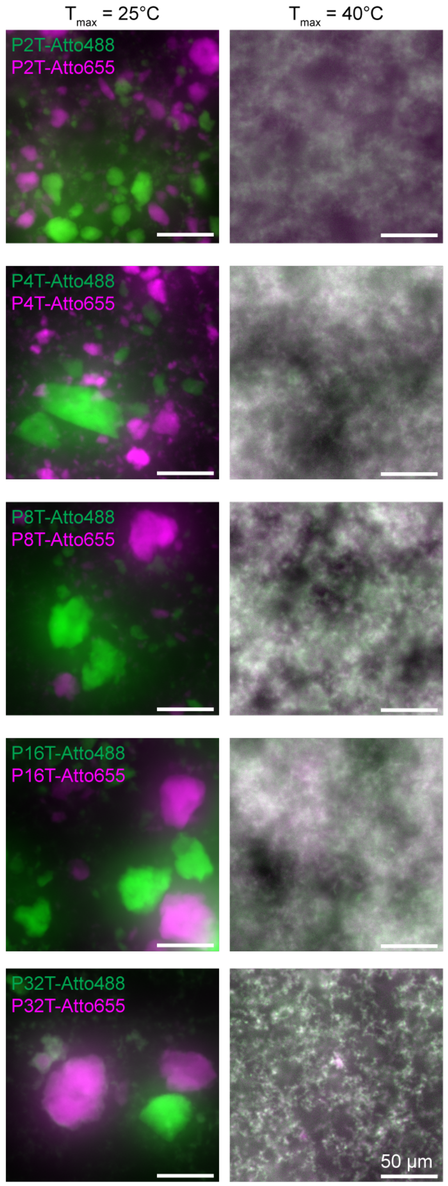

Figure S4: Fluorescence microscopy images of each Dipid variant, each containing a mix of structures labeled with Atto488 (green) or Atto655 (magenta), upon gradual cooling after incubation at the indicated temperatures T_{max} for 1 h. Scale bars: 50 µm.

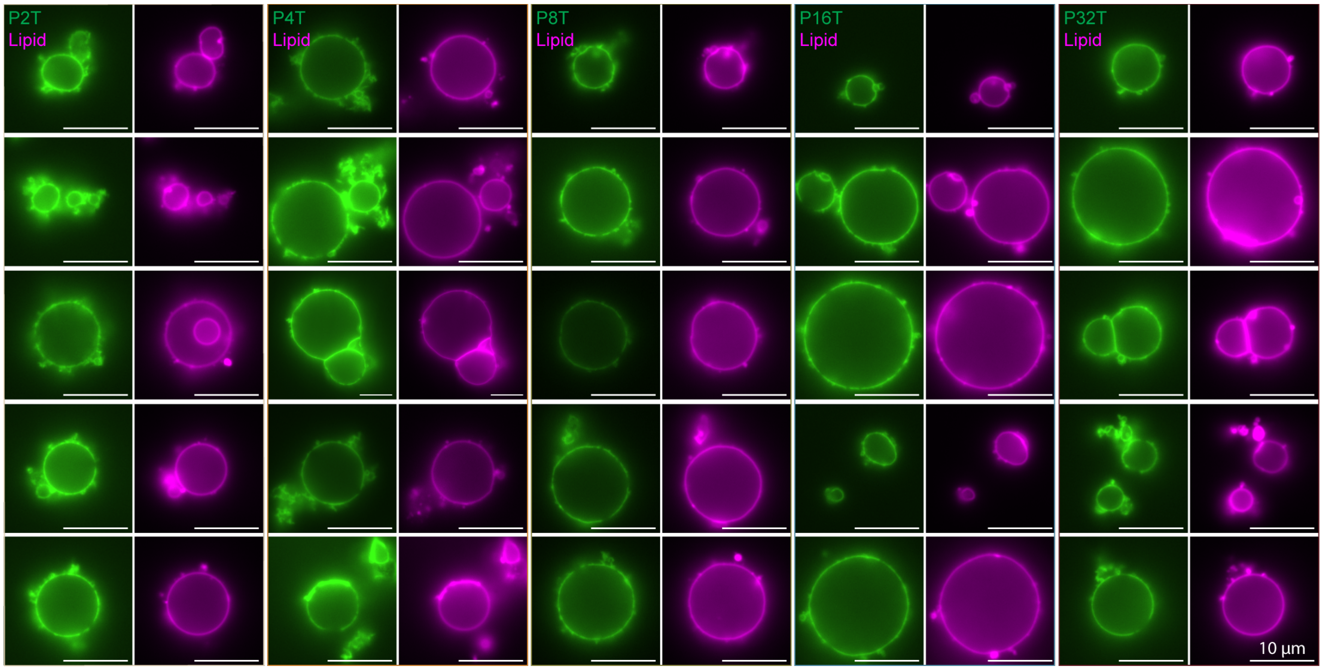

Figure S5: Fluorescence microscopy images of each Dipid variant (green), upon reassembly on GUVs (magenta), forming outer DNA shells. Scale bars: 10 µm.

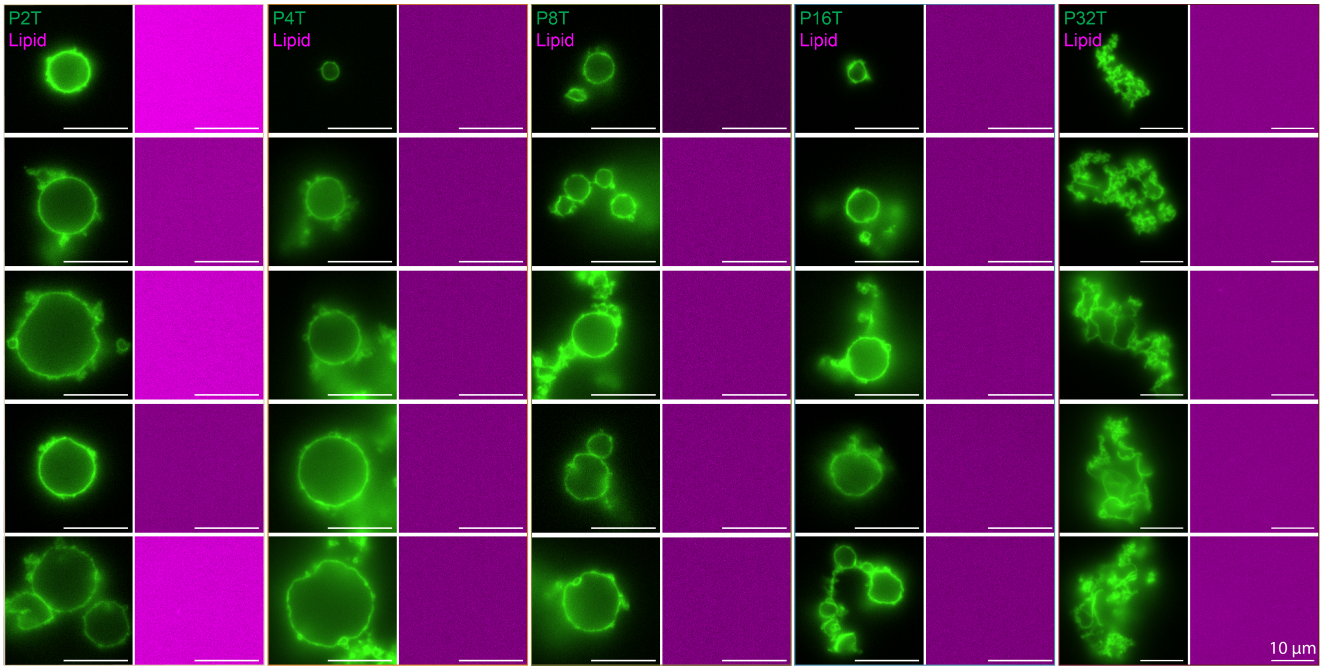

Figure S6: Fluorescence microscopy images of each Dipid variant (green), upon solubilization of GUVs (magenta) after vesicle-templated assembly, forming freestanding spherical Dipid shells with the exception of P32T, where Dipid shells collapsed. Scale bars: 10 µm.

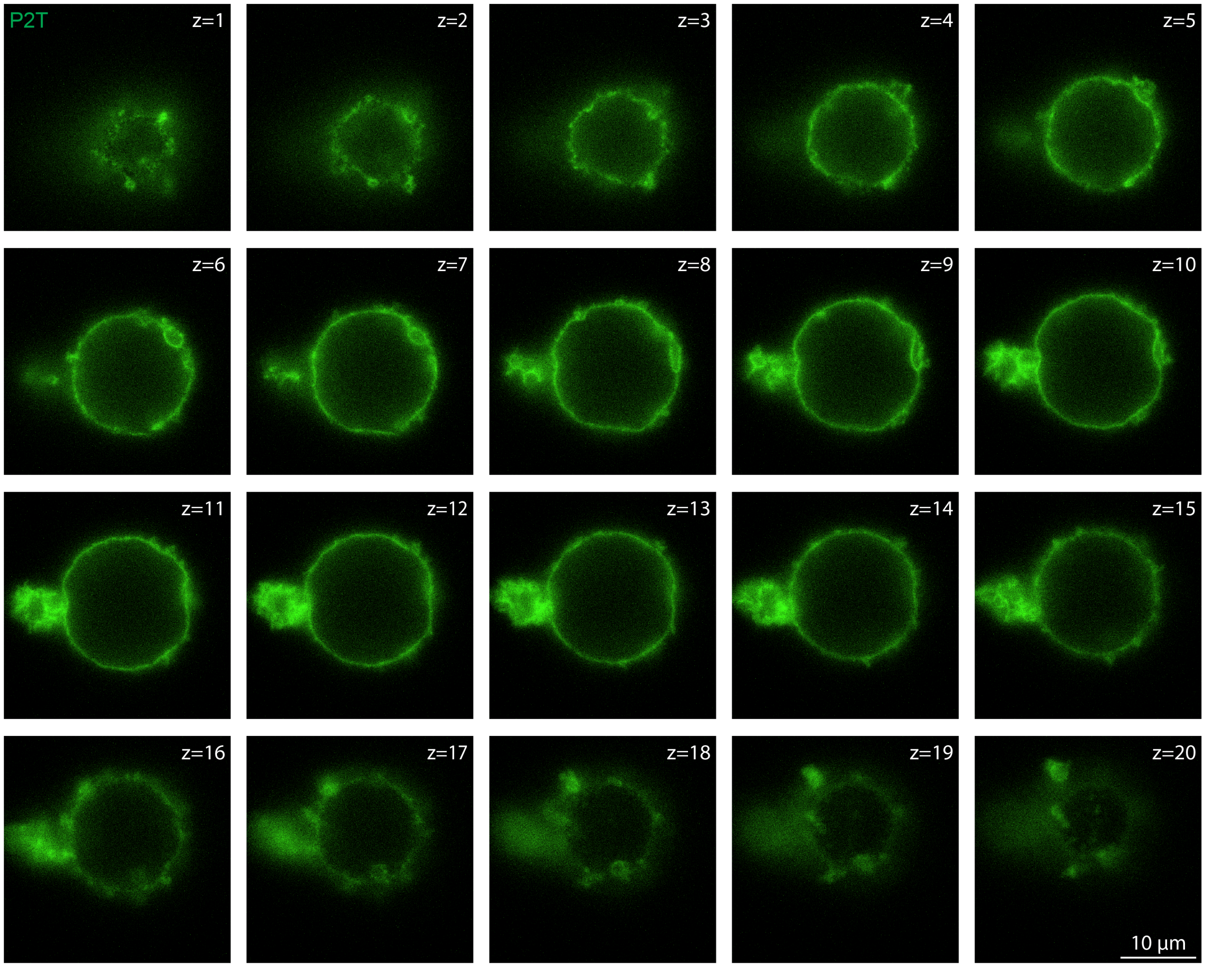

Figure S7: Fluorescence microscopy Z-slices of the freestanding Dipid shell (green) from Figure 3 ($\Delta z = 1\,\mu\text{m}$). Scale bar: $10\,\mu\text{m}$.

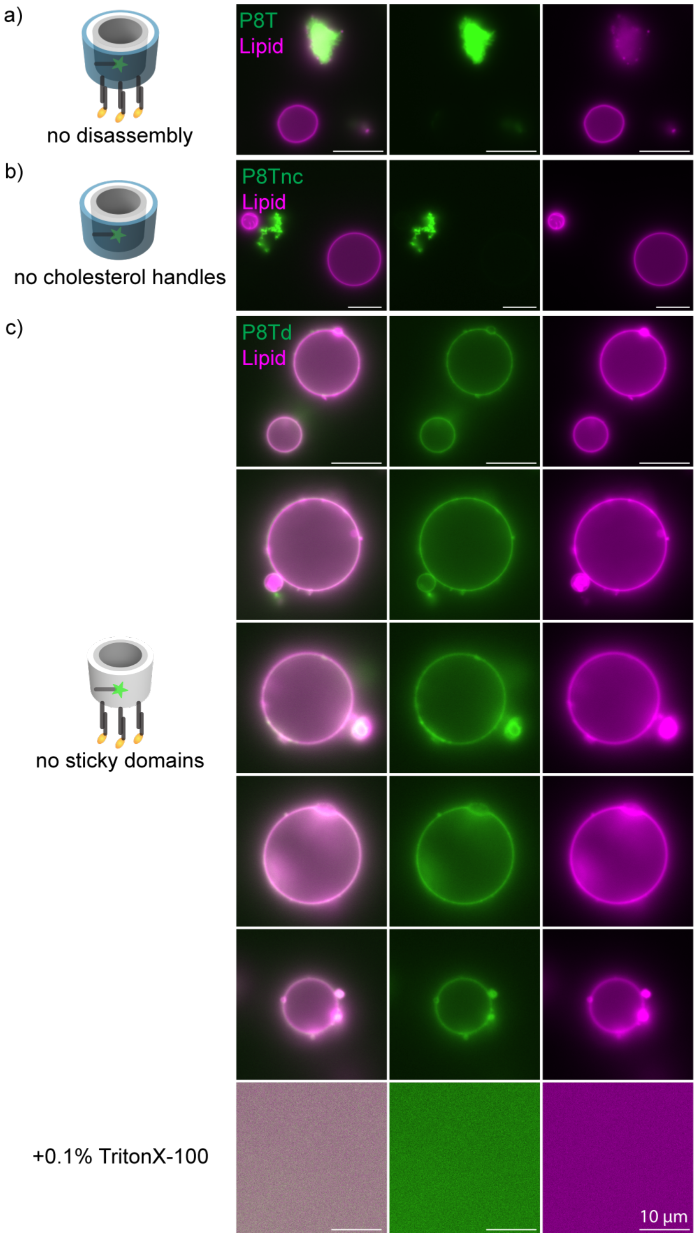

Figure S8: Fluorescence microscopy images showing no formation of Dipid shells (green) enclosing GUVs (magenta) when a) no disassembly occurs for P8T, or when b) cholesterol handles (dark gray rods) protruding from the bottom of the Dipid barrel are absent ("P8Tnc"), as well as no freestanding Dipid shells after Triton X–100 addition when c) sticky domains (blue cylinder layer) are absent ("P8Td") despite formation of outer Dipid shells. Scale bars: 10 µm.

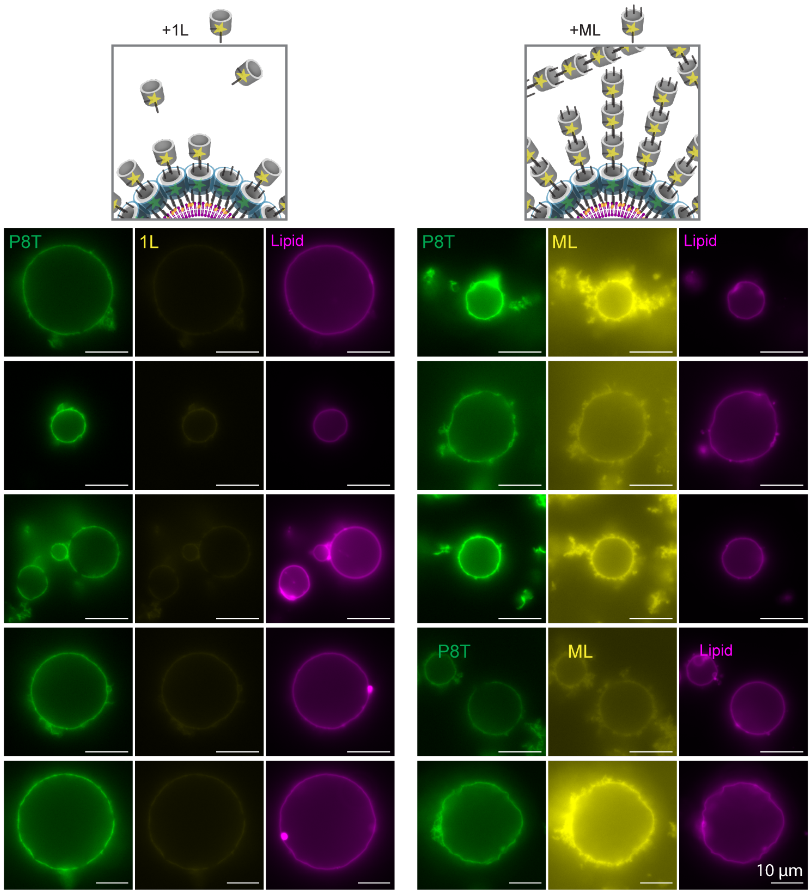

Figure S9: Schematics and corresponding fluorescence microscopy images of P8T Dipids (green), upon reassembly on GUVs (magenta), and subsequent addition of 1L and ML Dipids (yellow) forming bilayer and multilayer DNA shells. Scale bars: 10 µm.

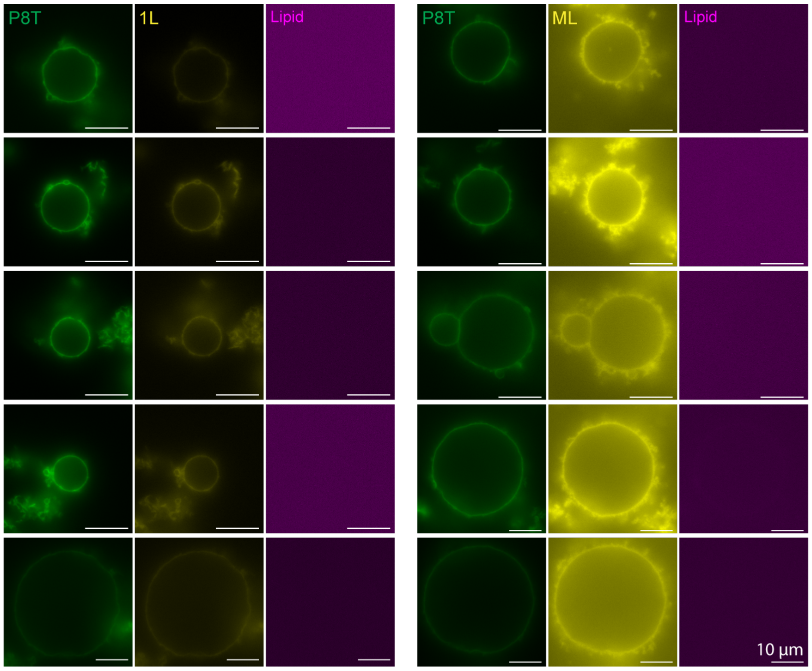

Figure S10: Fluorescence microscopy images of Dipids consisting of P8T (green) and 1L or ML (yellow), upon solubilization of GUVs (magenta) after vesicle-templated assembly, forming freestanding bilayer and multilayer spherical Dipid shells. Scale bars: 10 µm.

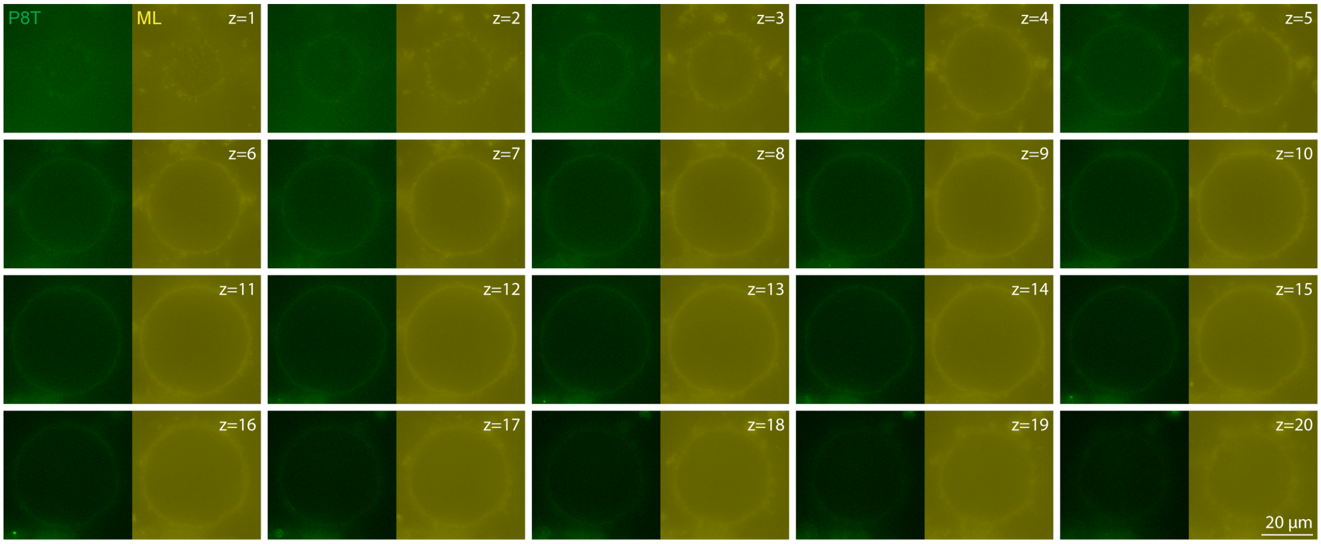

Figure S11: Fluorescence microscopy Z-slices of the freestanding multilayer Dipid shell consisting of P8T (green) and ML (yellow) from Figure 4c ($\Delta z = 2\,\mu\text{m}$). Scale bar: $20\,\mu\text{m}$.

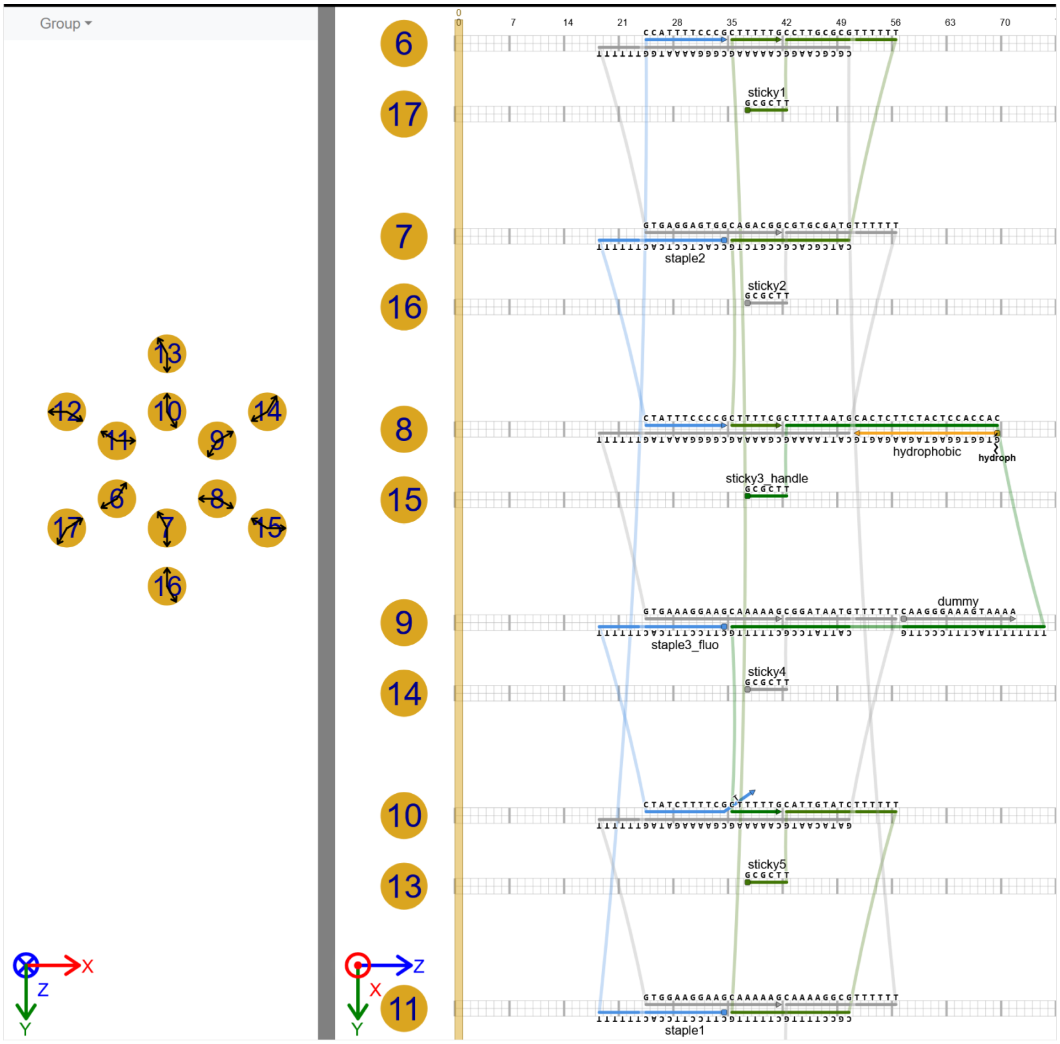

Figure S12: DNA strand routing of the minimal monomer in scadnano [6]. The design file is available on our GitHub repository.

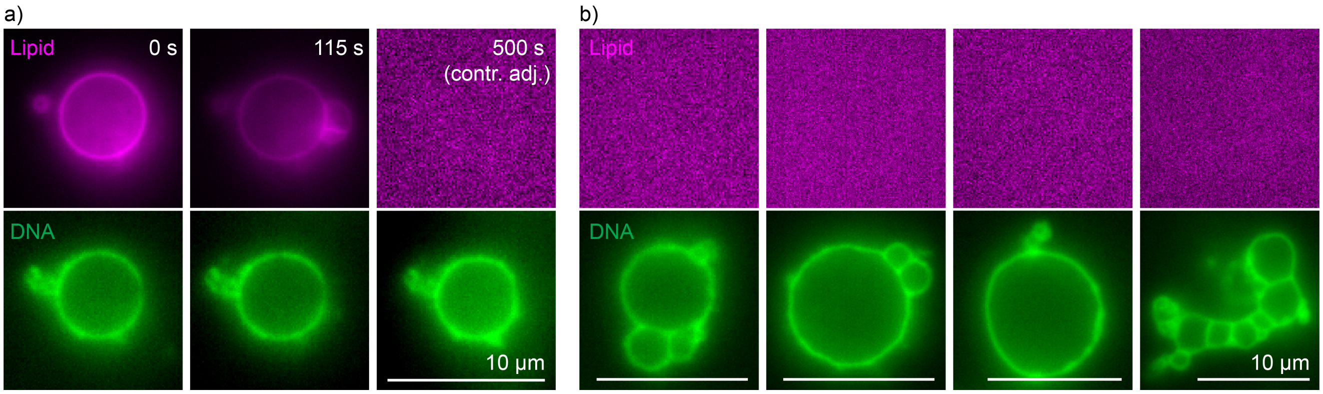

Figure S13: Freestanding DNA shells formed by the minimal monomer. a) Fluorescence time series showing the release of DNA shell (green) from GUVs (magenta). Triton X–100 was added to one side of a narrow channel containing DNA-enclosed GUVs and allowed to diffuse. $t = 0\,\mathrm{s}$ marks the start of acquisition. b) Freestanding DNA shells shown in Figure 4c, next to the lipid signal. Scale bars: 10 μm.

References

(1) Poppleton, E.; Matthies, M.; Mandal, D.; Romano, F.; Šulc, P.; Rovigatti, L. oxDNA: coarse-grained simulations of nucleic acids made simple. *Journal of Open Source Software* **2023**, *8*, 4693, DOI: 10.21105/joss.04693.

(2) Bohlin, J.; Matthies, M.; Poppleton, E.; Procyk, J.; Mallya, A.; Yan, H.; Šulc, P. Design and simulation of DNA, RNA and hybrid protein-nucleic acid nanostructures with oxView. *Nature Protocols* **2022**, *17*, 1762–1788, DOI: 10.1038/s41596-022-00688-5.

(3) Pettersen, E. F.; Goddard, T. D.; Huang, C. C.; Meng, E. C.; Couch, G. S.; Croll, T. I.; Morris, J. H.; Ferrin, T. E. UCSF ChimeraX: Structure visualization for researchers, educators, and developers. *Protein Science: A Publication of the Protein Society* **2021**, *30*, 70–82, DOI: 10.1002/pro.3943.

(4) Karfusehr, C.; Eder, M.; Yang, H. Y.; Beinsteiner, B.; Jasnin, M.; Simmel, F. C. Self-assembled cell-scale containers made from DNA origami membranes. *Nature Materials* **2025**, 1–9, DOI: 10.1038/s41563-025-02418-0.

(5) Wickham, S. F. J. et al. Complex multicomponent patterns rendered on a 3D DNA-barrel pegboard. *Nature Communications* **2020**, *11*, 5768, DOI: 10.1038/s41467-020-18910-x.

(6) Doty, D.; Lee, B. L.; Stérin, T. In *26th International Conference on DNA Computing and Molecular Programming (DNA 26)*, ed. by Geary, C.; Patitz, M. J., Schloss Dagstuhl – Leibniz-Zentrum für Informatik: Dagstuhl, Germany, 2020; Vol. 174, 9:1–9:17, DOI: 10.4230/LIPIcs.DNA.2020.9.

(7) Franquelim, H. G.; Khmelinskaia, A.; Sobczak, J.-P.; Dietz, H.; Schwille, P. Membrane sculpting by curved DNA origami scaffolds. *Nature Communications* **2018**, *9*, 811, DOI: 10.1038/s41467-018-03198-9.

(8) Schindelin, J. et al. Fiji: an open-source platform for biological-image analysis. *Nature Methods* **2012**, *9*, 676–682, DOI: 10.1038/nmeth.2019.

(9) Videbæk, T. E.; Hayakawa, D.; Hagan, M. F.; Grason, G. M.; Fraden, S.; Rogers, W. B. Measuring multisubunit mechanics of geometrically programmed colloidal assemblies via cryo-EM multi-body refinement. *Proceedings of the National Academy of Sciences* **2025**, *122*, e2500716122, DOI: 10.1073/pnas.2500716122.
